# Cellular localization of long non-coding RNAs affects silencing by RNAi more than by antisense oligonucleotides

**DOI:** 10.1093/nar/gkv1206

**Published:** 2015-11-17

**Authors:** Kim A. Lennox, Mark A. Behlke

**Affiliations:** Integrated DNA Technologies, Inc., Coralville, IA 52241, USA

## Abstract

Thousands of long non-coding RNAs (lncRNAs) have been identified in mammalian cells. Some have important functions and their dysregulation can contribute to a variety of disease states. However, most lncRNAs have not been functionally characterized. Complicating their study, lncRNAs have widely varying subcellular distributions: some reside predominantly in the nucleus, the cytoplasm or in both compartments. One method to query function is to suppress expression and examine the resulting phenotype. Methods to suppress expression of mRNAs include antisense oligonucleotides (ASOs) and RNA interference (RNAi). Antisense and RNAi-based gene-knockdown methods vary in efficacy between different cellular compartments. It is not known if this affects their ability to suppress lncRNAs. To address whether localization of the lncRNA influences susceptibility to degradation by either ASOs or RNAi, nuclear lncRNAs (MALAT1 and NEAT1), cytoplasmic lncRNAs (DANCR and OIP5-AS1) and dual-localized lncRNAs (TUG1, CasC7 and HOTAIR) were compared for knockdown efficiency. We found that nuclear lncRNAs were more effectively suppressed using ASOs, cytoplasmic lncRNAs were more effectively suppressed using RNAi and dual-localized lncRNAs were suppressed using both methods. A mixed-modality approach combining ASOs and RNAi reagents improved knockdown efficacy, particularly for those lncRNAs that localize to both nuclear and cytoplasmic compartments.

## INTRODUCTION

Advancements in genomics technologies over the last decade have led to the identification of tens of thousands of previously unsuspected long non-coding RNAs (lncRNAs), revolutionizing the way we view the complex eukaryotic transcriptome (1,2). While biological relevance for the majority of lncRNAs has not been assessed, those lncRNAs that have been functionally characterized are reported to play important roles in a variety of physiological processes, such as maintaining homeostasis, regulating cell growth and differentiation, apoptosis, imprinting, promoting pluripotency and controlling gene expression (3–8). Because of their varied roles in cellular processes, dysregulation of lncRNAs can contribute to several pathologic states including cancer and neurodegeneration (reviewed in (9,10)). In fact, thanks to their aberrant expression pattern in many cancer cells, lncRNAs can be excellent tumor biomarkers and may be diagnostic and/or prognostic for some tumor types (11–13).

LncRNAs comprise a remarkably diverse population of cellular RNAs. By definition, these are non-protein coding RNAs longer than 200 nt. Length varies from the small 7Sk species (∼330 nt) to the very large Airn (∼118 kb). LncRNAs can be transcribed from intergenic, intragenic or intronic regions. Further, these species can be spliced or remain unspliced and are often 5′-capped and 3′-polyadenylated (reviewed in (14)). Through means of their sequence or secondary structure formation, lncRNAs can interact with or bind to other cellular nucleic acids or proteins. Importantly, lncRNAs can shuttle to various subcellular locations. Some show markedly different levels of accumulation in the nucleus versus the cytoplasm while others are equally distributed between both compartments (13,15,16).

Methods to degrade cellular RNAs using synthetic oligonucleotides have been used for many years as an approach to study gene function. The present study employs both ‘antisense’ and RNA interference (RNAi) approaches. ‘Antisense methods’ were first used to suppress gene expression over 35 years ago (17–19) and employ synthetic oligonucleotides (antisense oligonucleotides or ASOs) that bind the target RNA and trigger degradation by endogenous RNase H, an enzyme that cleaves the RNA strand in a DNA/RNA heteroduplex. Two variants of RNase H exist in mammalian cells: RNase H1 and RNase H2. It is believed that RNase H1 is responsible for ASO-directed RNA degradation (20–24). ASOs can also be used as ‘steric-blocking’ agents where tight binding of the ASO to the target RNA interferes with function in the absence of degradation (25); however, this approach is not employed in the present study.

Unmodified DNA oligonucleotides were the first compounds used for antisense knockdown (26); however, these compounds are rapidly degraded in serum and the intracellular environment and thus have poor efficacy (27,28). A variety of design and chemical modification strategies have evolved to improve the functionality of ASOs (29). DNA oligonucleotides with a phosphorothioate (PS) backbone represented the first improvement in ASO design; DNA-PS ASOs have substantial nuclease resistance yet retain the ability to trigger RNase H. Unfortunately, the PS modification also decreases the melting temperature (*T*_m_) of the oligonucleotide, which can lower potency. A chimeric ASO design was developed that employs *T*_m_-enhancing modifications at the ends to increase binding affinity to the target, retaining a central DNA core to form a substrate for RNase H when duplexed with the RNA target (termed a ‘gapmer’) (30,31). The *T*_m_-enhancing modifications most commonly used include a spectrum of chemical groups substituted at the 2′ position of the ribose backbone, such as 2′-*O*-Methyl (2′ OMe) RNA and locked nucleic acids (LNA) (32). ASOs combining DNA, PS linkages and either 2′ OMe or LNA flanking domains in the form of gapmer compounds can be very potent (19,33,34).

In recent years, RNA interference (RNAi) has gained favor in the research community as a primary tool to reduce mRNA levels. RNAi is a natural process to regulate gene expression that exists in most eukaryotic cells. RNAi employs small, double-stranded RNA molecules that associate with multiple protein factors to form the RNA-Induced Silencing Complex (RISC), leading to either suppression of translation or degradation of a target mRNA. Natural RNAi effector molecules either arise from processing of endogenous hairpin transcripts by the nucleases Drosha and Dicer (microRNAs or miRNAs) or from long double-stranded RNAs (dsRNAs) processed by Dicer alone (small interfering RNAs or siRNAs) (35). Synthetic siRNA or miRNA mimics can be made from oligonucleotides and used to suppress gene expression. The canonical siRNA trigger is a 21mer dsRNA with 2-base 3′ overhangs. When bound by Argonaut 2 (AGO2) in RISC, the ‘passenger’ strand is discarded, leaving the ‘guide’ (active) strand in single-stranded form, free to bind an RNA target that is subsequently cleaved (36). Another strategy is to use longer, 27mer asymmetric double-stranded RNAs that are Dicer substrates (DsiRNAs). Engaging Dicer processing can increase the potency of the 27mer DsiRNAs when compared to the 21mers, perhaps due to effects relating to Dicer involvement with RISC formation and siRNA loading into AGO2 (37–39). Chemical modifications, such as 2′ OMe RNA, 2′-fluoro RNA and LNA residues, can be incorporated into siRNAs to improve stability, evade detection by the innate immune system and mitigate off-target effects (40).

Although both RNAi and antisense methods have already been successfully used to knockdown specific lncRNAs (41–45), the relative effectiveness of each method might not be the same for all lncRNAs. Of particular concern, the relative abundance of the protein factors that mediate RNA degradation vary between cellular compartments and may influence the relative ability of these tools to suppress a lncRNA, depending on its location. Given that its primary function is to assist in removing RNA from genomic DNA following replication, RNase H1 is usually thought to primarily be a nuclear enzyme. Nevertheless, some level of RNase H1 activity is found in the cytoplasm (21,46) and a specific variant of RNase H1 localizes to mitochondria (47). RNase H active ASOs are known to be capable of targeting intronic sequences and the efficacy of this approach is influenced by splicing efficiency (48). Further, steric blocking antisense approaches have been used for many years as a method to alter splicing in the nucleus by blocking access of splice acceptor/donor sequences in introns (splice-switching oligonucleotides or SSOs) (49). Different forms of nuclear RNAi have been described that vary both in function and protein composition (45,50). In some reports, the RISC variant that degrades RNA (AGO2-RISC) was found to be primarily cytoplasmic and active mRNA cleavage was greatest at the rough endoplasmic reticulum (22,51–55). It has also been demonstrated that RNAi is ineffective at degrading targeted intronic sequences or RNAs engineered to be retained in the nucleus (22,53); siRNAs have also been reported to be non-functional against nuclear-localized snoRNAs and scaRNAs, suggesting reduced degradative RNAi activity in the nucleus (43,56,57). Contrary to this evidence, the degradative RNAi protein AGO2 has been detected in the nucleus (45,58–61) and reports show that the nuclear 7SK RNA can successfully be silenced by siRNAs (62,63). The ambiguity in the reported nuclear RNAi knockdown results may be due to nuclear RNA inaccessibility to nuclear RNAi components (sub-compartmentalization) or from protein binding which may vary between compartments. It therefore appears that although the protein factors that mediate degradative antisense and degradative RNAi exist in both the cytoplasm and the nucleus, their relative abundance, level of functional activity or ease of access varies between these two cellular compartments.

We investigated if the intracellular localization of individual lncRNAs influences which knockdown method is optimal to employ. We also compared different ASO and RNAi reagent designs and chemical modification strategies to determine if a specific reagent showed any particular advantage in this application. A set of seven lncRNAs with various cellular distribution patterns was selected from the literature to perform a comparative survey (Table 1). MALAT1 and NEAT1 are lncRNAs found predominantly in the nucleus; DANCR and OIP5-AS1 are found primarily in the cytoplasm; TUG1, CasC7 and HOTAIR have both nuclear and cytoplasmic distribution. These seven lncRNA targets were compared for efficiency of knockdown using different ASO and RNAi triggers (Table 2). LncRNA levels could be suppressed by each class of knockdown reagent tested. However, the relative efficiency of knockdown varied dramatically between targets and a correlation was observed between the subcellular localization of the target and the relative effectiveness of the antisense versus RNAi reagents. LncRNAs localized primarily in the nucleus (MALAT1 and NEAT1) were easier to target with ASOs. Conversely, lncRNAs residing in the cytoplasm (DANCR and OIP5-AS1) were more easily suppressed using RNAi reagents. LncRNAs distributed in both nuclear and cytoplasmic compartments (TUG1, CasC7 and HOTAIR) were suppressed by both knockdown strategies, although overall the ASOs outperformed the RNAi reagents. We found that combining ASOs and RNAi reagents could have additive effects, particularly for the dual-localized targets. Similar additive effects were previously reported for mRNAs (64). This may be an attractive strategy to employ, especially for dual-localized lncRNAs or if the subcellular distribution is unknown.

**Table 1.** LncRNAs with various subcellular distributions selected for knockdown studies

LncRNA	NCBI ID	Ensemble ID	Length	Location
MALAT1	NR_002819	ENSG00000251562	8708	Nucleus
NEAT1	NR_028272	ENSG00000245532	3756	Nucleus
DANCR	NR_024031	ENSG00000226950	855	Cytoplasm
OIP5-AS1	NR_026757	ENSG00000247556	1894	Cytoplasm
TUG1	NR_002323	ENSG00000253352	7542	Mixed
CasC7	HG501752.1	ENSG00000259758	9346	Mixed
HOTAIR	NR_047518	ENSG00000228630	2337	Mixed

The transcript lengths were determined from the NCBI ID data.

**Table 2.** Chemical modification schematic for oligonucleotides employed in the knockdown studies

**Oligonucleotide design examples**
**DNA-PS ASO:**
D*D*D*D*D*D*D*D*D*D*D*D*D*D*D*D*D*D*D*D
**2′OMe-PS ASO:**
M*M*M*M*M*D*D*D*D*D*D*D*D*D*D*M*M*M*M*M
**LNA-PS ASO:**
L*L*L*D*D*D*D*D*D*D*D*D*D*L*L*L
**siRNA (21mer):**
5′ RRRRRRRRRRRRRRRRRRRRR 3′
3′ RRRRRRRRRRRRRRRRRRRRR 5′
**DsiRNA (27mer):**
5′ RRRRRRRRRRRRRRRRRRRRRRRDD 3′
3′ RRRRRRRRRRRRRRRRRRRRRRRRRRR 5′

D = DNA; M = 2′OMe; L = LNA; R = RNA; ‘*’ = PS linkage.

Placement of the LNA modifications in the LNA-PS ASO are proprietary to Exiqon and may vary with ASO sequence; the 3–10–3 design shown is for illustrative purposes only.

## MATERIALS AND METHODS

### Experimental design and site selection

Three design variants were tested for both the ASO and RNAi arms of the study (Table 2). Reagents were designed to target the seven lncRNA species listed in Table 1 as follows. For each lncRNA, a set of 20mer DNA-PS and 2′ OMe-PS ASOs were synthesized at the same 12 sites. These sites were selected to lie in areas predicted to have low secondary structure in each lncRNA based on UNAfold software output (The RNA Institute, Albany, NY, USA). Within these ‘accessible areas’, sites of 20 nt length were screened for the following criteria: 40–60% GC content, minimal hairpin potential, low self-dimerization potential and at least 4 out of 20 base mismatch on a BLASTn search of the human transcriptome. Additionally, a set of six LNA™ longRNA GapmeRs were designed for each target using proprietary software available on the Exiqon website (Vedbaek, Denmark). Due to the different approaches/algorithms employed, the LNA-PS ASOs targeted different sites than the DNA-PS and 2′ OMe-PS ASOs. For the RNAi reagents, unmodified 21mer siRNA sites were selected using the online GE Healthcare design algorithm (Dharmacon, Lafayette, CO, USA). Sites for 27mer DsiRNAs were selected using an internal Integrated DNA Technologies algorithm (Integrated DNA Technologies (IDT), Coralville, IA, USA). The 21mer siRNA and 27mer DsiRNA design algorithms were optimized for their respective compounds and the sites selected were different between reagents for each target. In addition, four LNA-modified 21mer siRNAs (Silencer^®^ Select siRNAs) were designed using the Ambion site selection algorithm (Thermo Fisher Scientific, Waltham, MA, USA).

### Oligonucleotide reagents

Antisense oligonucleotides (DNA-PS and 2′ OMe-PS gapmers), primers and probes were chemically synthesized using standard phosphoramidite chemistry. RNA oligonucleotides (21mer unmodified siRNAs and 27mer DsiRNAs) were chemically synthesized using t-Butyl-dimethylsilyl (TBDMS) chemistry (Integrated DNA Technologies). Probes for qPCR were purified using reversed phase high performance liquid chromatography (RP-HPLC) (Integrated DNA Technologies) while all other oligonucleotides were prepared as sodium salts. All oligonucleotides were analyzed by electrospray-ionization mass spectrometry (ESI-MS) and were within ±0.02% predicted mass. Oligonucleotide concentrations were calculated using modification-specific extinction coefficients based on measured ultraviolet (UV) absorbance at 260 nm. RNA duplexes were annealed in IDT duplex buffer (30 mM HEPES, pH 7.5, 100 mM Potassium Acetate) (Integrated DNA Technologies). Silencer^®^ Select siRNAs were purchased from Thermo Fisher Scientific (Waltham, MA, USA) and LNA™ longRNA GapmeRs were purchased from Exiqon (Vedbaek, Denmark). All oligonucleotide sequences are shown in Supplementary Table S1 in the online Supplemental Data.

### Cell culture and transfections

To compare the knockdown efficiency between reagents, oligonucleotides were transfected in biological triplicate into HeLa cells with Lipofectamine^®^ 2000 at 10, 3 and 1 nM doses. HeLa cells were cultured in ATCC recommended media. For transfections (optimized for this system), oligonucleotides were reverse transfected in 96-well plates by complexing the various oligonucleotide doses with 0.5 μl Lipofectamine^®^ 2000 (Thermo Fisher Scientific) in OptiMEM^®^ I (Thermo Fisher Scientific) for a total volume of 50 μl in each well. HeLa cells (20 000) were suspended in 100 μl Dulbecco's Modified Essential Medium (DMEM) containing 10% fetal bovine serum (Thermo Fisher Scientific), added to the lipid-oligonucleotide complexes, then incubated for 24 h at 37°C and 5% CO_2_. For HOTAIR, transfections were performed in 48-well plates with 40 000 HeLa cells per well, with doubling of all reagent volumes. Each 96-well transfection plate included a positive transfection control (DsiRNA, 2′ OMe-PS ASO or LNA-PS ASO targeting HPRT) and two or three compound-specific negative transfection controls. All transfections were performed in biological triplicate and each experiment was performed at least twice. The results of all transfections performed were averaged.

### RNA isolation and RT-qPCR

LncRNA knockdown was measured by reverse transcription quantitative PCR (RT-qPCR), comparing each knockdown reagent with the appropriate chemically modified negative control oligonucleotide at the same dose and calculated from an average of two or three separate experiments. RNA was isolated 24 h after transfection with the SV 96 Total RNA Isolation Kit (Promega, Madison, WI, USA) with DNase1 treatment. cDNA was synthesized from ∼200 ng total RNA with anchored oligonucleotide dT and random hexamer primers (Integrated DNA Technologies) using SuperScript^®^ II Reverse Transcriptase (Thermo Fisher Scientific) per the manufacturer's instructions. qPCR reactions were performed using ∼10 ng cDNA with Immolase DNA polymerase (Bioline, Randolph, MA, USA), 500 nM of each primer and 250 nM probe in 10 μl reactions in 384-well plate format. Amplification reactions were run on an Applied Biosystems 7900HT (Thermo Fisher Scientific) with cycling conditions consisting of enzyme activation at 95°C for 10 min, followed by 40 cycles of 2-step PCR (95°C for 15 s, 60°C for 1 min). All qPCR reactions were performed in triplicate and averaged. Linearized cloned amplicons were used as copy number standards to establish absolute quantitative measurements for each assay. LncRNA expression levels were quantified by multiplexing two 5′-nuclease assays per target (one assay located toward the 5′ end and one located toward the 3′ end of the transcript) and normalized against both HPRT (NM_000194) and SFRS9 (NM_003769) as internal reference controls. LncRNA knockdown levels were calculated by comparing cells transfected with ASO or RNAi reagents to cells transfected with appropriate chemical modification-specific negative control oligonucleotides. Data are plotted graphically with error bars representing standard error of the mean (SEM).

### IC50 calculations

HeLa and HCT116 cells were transfected in biological triplicates with ASOs or siRNAs as previously described. A range of nine doses for each knockdown reagent was selected based on empirically determined oligonucleotide activity and tested in both cell lines. RNA extraction, cDNA synthesis and qPCR reactions were performed as previously described. IC50 values were calculated with GraphPad Prism^®^ 6.0 software using the non-linear regression formula: log (inhibitor) versus normalized response—variable slope (Graphpad Software Inc, San Diego, CA, USA).

## RESULTS

### Nuclear target knockdown: MALAT1 and NEAT1

MALAT1 (metastasis associated in lung adenocarcinoma transcript 1) is a highly abundant ∼8.7 kb lncRNA that localizes to nuclear speckles and plays a role in both regulating alternative splicing and cellular proliferation (65–67). MALAT1 is associated with tumor metastasis and is overexpressed in several human carcinomas (66,68–70). NEAT1 (Nuclear Enriched Abundant Transcript 1, ∼3.7 kb) is a lncRNA localized in nuclear paraspeckles and is thought to be involved in regulating transcription within these subnuclear bodies (71–74). NEAT1 is upregulated in Huntington's disease and involved in HIV replication (75,76). Both ASOs and siRNAs have been successfully used in knockdown experiments targeting MALAT1 and NEAT1 (24,45,65,68,70–72,76–78). MALAT1 ASO site 5042 was previously shown to suppress MALAT1 expression using a 2′-*O*-methoxyethyl phosphorothioate gapmer ASO (ISIS395254) (79).

**Figure 1.**
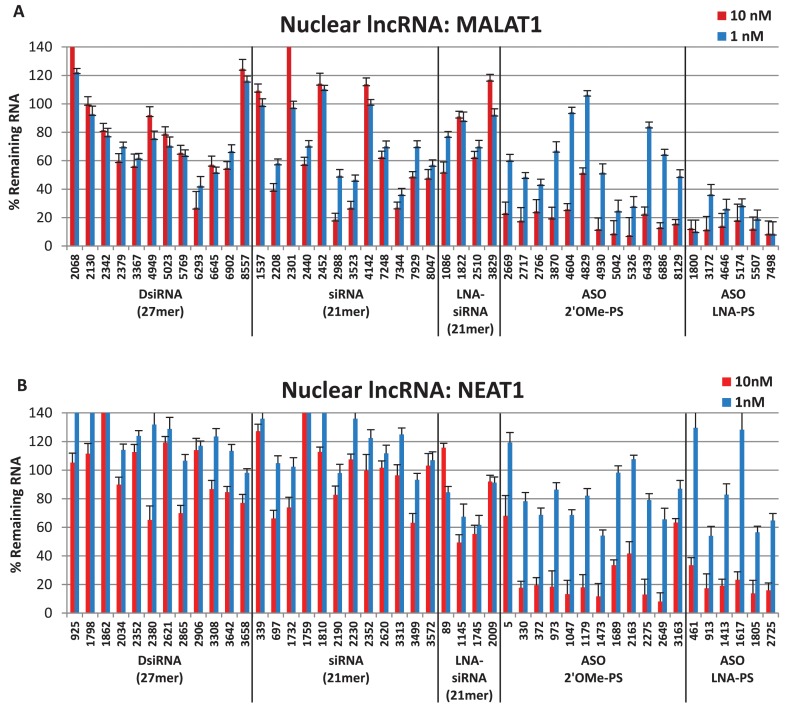
Nuclear lncRNA knockdown. DsiRNAs, siRNAs and ASOs targeting (**A**) MALAT1 or (**B**) NEAT1 were transfected in triplicate into HeLa cells with Lipofectamine^®^ 2000, and RNA levels were measured by RT-qPCR 24 h later. Data shown represent an average of two or more experiments (at least six independent transfections). LncRNA levels were calculated using the internal reference genes HPRT and SFRS9, and compared with HeLa cells transfected with 2–3 negative control sequences containing the same chemical modifications at the same doses. Site locations in the target are indicated on the x-axis and are organized 5′ to 3′ along each target for each class of knockdown reagent.

Results are shown for MALAT1 and NEAT1 (Figure 1A and B) with ASO and RNAi sites linearly ordered along the transcript from the 5′- to the 3′-end. As expected from prior experience with mRNA knockdown, the 2′ OMe-PS gapmer ASOs showed higher efficacy over the DNA-PS ASOs at the same sites (19,33). To unclutter data presentation, only results for the 2′ OMe-PS ASOs are included in the manuscript figures. Plots comparing activity of DNA-PS and 2′ OMe-PS ASOs at all sites in all targets are shown in the online Supplemental Data (Supplementary Figures S1–S7). Two RT-qPCR assays were employed to measure RNA levels for each target. Results from a single assay are shown. In general, RNA levels were grossly concordant between assays. Results from both RT-qPCR assays for the MALAT1 screen are shown in Supplementary Figure S8 in the online Supplementary Data.

For the two nuclear lncRNAs studied, the ASOs were consistently more effective at suppressing RNA levels than were the RNAi reagents. For MALAT1 (Figure 1A), several of the RNAi reagents reduced RNA levels by ∼60–70% at 10 nM dosing, but overall ASO performance was superior, resulting in higher levels of suppression (as high as 80–85%). An LNA gapmer ASO was the most potent reagent of the 58 compounds tested. In general, no appreciable difference in effectiveness was observed between the different classes of RNAi reagents (i.e. DsiRNAs versus siRNAs versus LNA-siRNAs). For NEAT1 (Figure 1B), the disparity between RNAi knockdown and ASO knockdown was even greater, with the best siRNA reducing NEAT1 levels by ∼50% while 12 of the ASOs tested achieved 80% knockdown or better.

To verify that the RNAi reagents and ASOs did not cause non-specific inhibition of nuclear lncRNAs, all of the MALAT1 or NEAT reagents were individually transfected into HeLa cells as previously described and RNA levels for both MALAT1 and NEAT1 RNA were measured. None of the MALAT1 knockdown reagents affected NEAT1 levels (Supplementary Figure S9A), and none of the NEAT1 knockdown reagents affected MALAT1 levels (Supplementary Figure S9B), demonstrating target specificity.

### Cytoplasmic target knockdown: DANCR and OIP5-AS1

DANCR (Differentiation Antagonizing Non-Protein Coding RNA) is a small ∼0.8 kb lncRNA involved in maintaining the undifferentiated state of certain cell types in the epidermis and regulating osteoblast differentiation; earlier reports indicated that this target can be suppressed using either shRNAs or siRNAs (15,80–82). RNA-FISH analysis showed that DANCR (or ANCR) heavily populates the cytosol, while RNA-seq analysis demonstrated that DANCR predominately clusters in the 40S/60S ribosomal cytosolic fraction (15,16). The second cytoplasmic lncRNA targeted in this study was the ∼1.9 kb OIP5-AS1 (OIP5 antisense RNA 1). The function of this lncRNA is currently unknown in humans, but its zebrafish ortholog, Cyrano, is involved in brain and eye development. Knockdown of Cyrano in zebrafish using steric-blocking phosphorodiamidate morpholino ASOs causes a reduction in head and eye size and neural tube opening defects, all of which could be rescued with ectopic expression of the human ortholog, OIP5-AS1 (83). As with DANCR, RNA-seq analysis of OIP5-AS1 showed that it clusters with the 40S/60S rRNA fraction and is predominantly localized in the cytoplasm (15).

**Figure 2.**
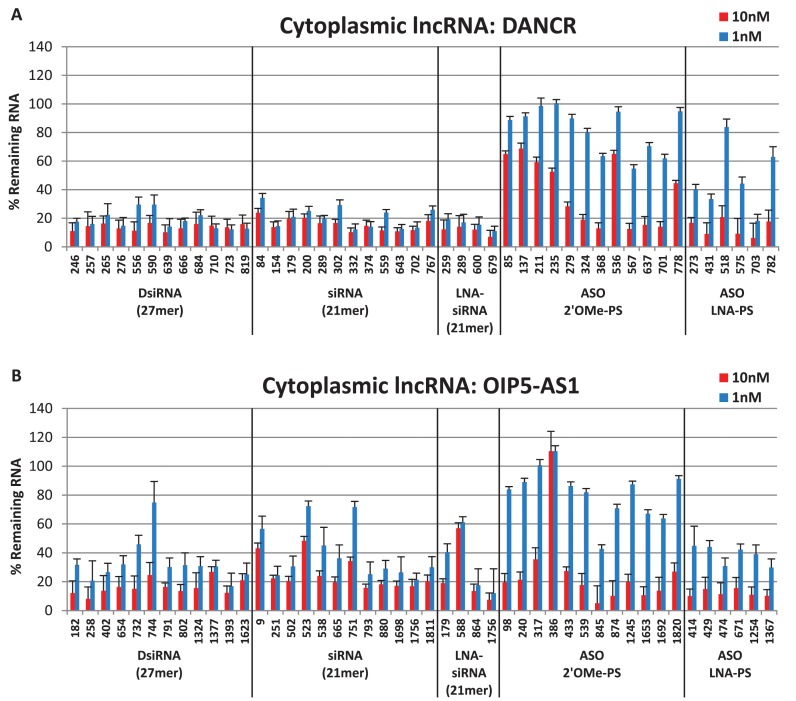
Cytoplasmic lncRNA knockdown. DsiRNAs, siRNAs and ASOs targeting (**A**) DANCR or (**B**) OIP5-AS1 were transfected in triplicate into HeLa cells with Lipofectamine^®^ 2000, and RNA levels were measured by RT-qPCR 24 h later. Data shown represent an average of two or more experiments (at least six independent transfections). LncRNA levels were calculated using the internal reference genes HPRT and SFRS9 and compared with HeLa cells transfected with 2–3 negative control sequences containing the same chemical modifications at the same doses. Site locations in the target are indicated on the x-axis and are organized 5′ to 3′ along each target for each class of knockdown reagent.

The cytoplasmic lncRNA DANCR was effectively suppressed by all 28 RNAi reagents tested, of which 17 out of 28 showed 80% or higher reduction in DANCR RNA levels at the 1 nM dose (Figure 2A). While some of the ASOs were also effective in reducing DANCR expression at the 10 nM dose, only a single LNA-PS ASO led to 80% reduction in DANCR RNA levels at the 1 nM dose. The cytoplasmic lncRNA OIP5-AS1 was also suppressed more effectively by the RNAi reagents (Figure 2B). While many of the ASOs were effective at the 10 nM dose, they were noticeably less potent at the 1 nM dose than the majority of RNAi reagents. Thus, the cytoplasmic lncRNAs studied showed superior knockdown using RNAi than antisense reagents, the exact opposite pattern seen for the nuclear lncRNAs studied.

### Dual-localized target knockdown: TUG1, CasC7 and HOTAIR

The first dual-localized target studied was TUG1 (Taurine upregulated Gene 1), a ∼7.5 kb lncRNA necessary for retinal development and involved in the upregulation of growth-control genes (15,16,84–86). TUG1 expression is also elevated in Huntington's disease as well as urothelial carcinoma of the bladder (75,87) and successful knockdown of TUG1 with siRNAs has been reported (84–87). CasC7 (cancer susceptibility candidate 7 or long intergenic non-protein coding RNA 980) is a ∼9.3 kb lncRNA whose function is currently unknown. This dual-localized lncRNA is present in nuclear fractions, yet also associates with large, cytoplasmic polysomal complexes, the latter of which implicates CasC7 in translational regulation (15). The final lncRNA surveyed in this study was HOTAIR (HOX transcript antisense RNA), a ∼2.3 kb transcript which is localized in both the nucleus and cytoplasm (84). HOTAIR has been shown to interact with the Polycomb Repressive Complex 2 (PRC2) to down-regulate the expression of multiple targeted genes (88,89). HOTAIR expression levels correlate with enhanced tumor metastasis and can serve as a negative prognostic marker for breast, colon, liver, esophageal squamous cell and pancreatic cancer patients (88–94). Knockdown of HOTAIR has been achieved with RNAi both *in vitro* and *in vivo*, and Bhan *et al*. inhibited HOTAIR with a DNA-PS ASO (88,89,91,93–98).

**Figure 3.**
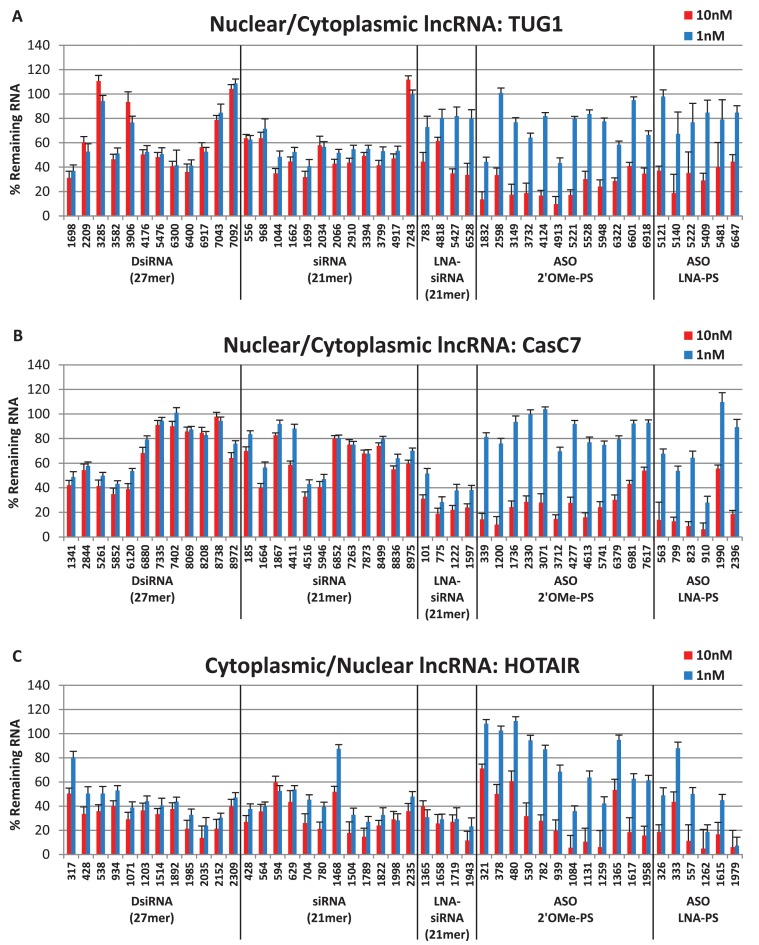
Dual-localized lncRNA knockdown. DsiRNAs, siRNAs and ASOs targeting (**A**) TUG1, (**B**) CasC7 or (**C**) HOTAIR were transfected in triplicate into HeLa cells with Lipofectamine^®^ 2000, and RNA levels were measured by RT-qPCR 24 h later. Data shown represent an average of two or more experiments (at least six independent transfections). LncRNA levels were calculated using the internal reference genes HPRT and SFRS9, and compared with HeLa cells transfected with 2–3 negative control sequences containing the same chemical modifications at the same doses. Site locations in the target are indicated on the x-axis and are organized 5′ to 3′ along each target for each class of knockdown reagent.

Both ASO and RNAi reagents were able to reduce TUG1 expression (Figure 3A), although higher levels of suppression were achieved using the ASOs. The best DsiRNAs and siRNAs each led to ∼70% reduction in TUG1 RNA levels while the best 2′ OMe-PS ASO and LNA-PS ASO led to 90% or 80% reductions in TUG1 RNA levels, respectively. Similarly, the antisense reagents outperformed the RNAi reagents for suppression of CasC7 RNA levels (Figure 3B). The best performing RNAi agent was an LNA-modified siRNA that reduced CasC7 levels by 80%, while three of the ASOs (one 2′ OMe-PS and two LNA-PS) reduced CasC7 levels by more than 90%. RNAi and antisense reagents were both able to reduce HOTAIR RNA (Figure 3C); however, only 1 out of 28 RNAi sites showed 90% suppression while 6 out of 18 ASO sites had 90% or higher suppression. In aggregate, the two classes of knockdown reagents showed more similar levels of performance when targeting lncRNAs with mixed cytoplasmic and nuclear distribution; however, in all three cases the overall highest levels of knockdown were achieved using antisense reagents.

**Figure 4.**
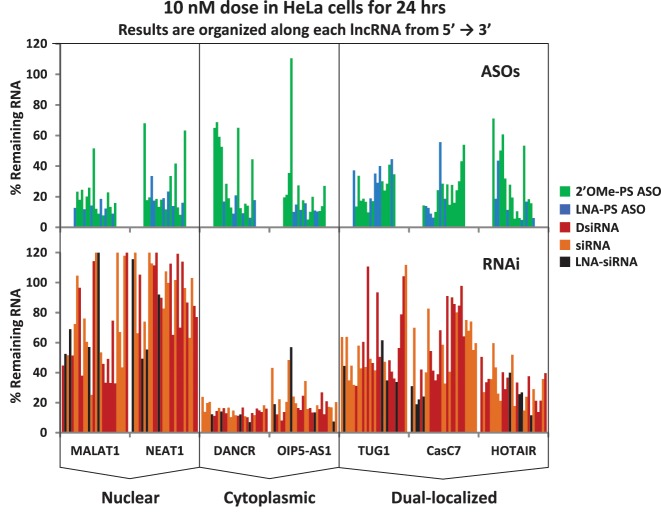
Data summary of lncRNA knockdown studies at 10 nM. Data are shown for all lncRNAs and all knockdown reagents for the 10 nM dose. The top panels comprise ASO knockdown data while the bottom panels comprise RNAi knockdown data, with the corresponding lncRNA name indicated at the bottom of the plots. For each class of compound, lncRNA targeted sites are linearly organized from left to right on the x-axis according to their 5′ to 3′ positions along the transcript. Each reagent type is indicated by color code.

A summary compilation of the knockdown studies performed on all seven lncRNAs (MALAT1, NEAT1, DANCR, OIP5-AS1, TUG1, CasC7 and HOTAIR) at the 10 nM dose is shown in Figure 4. Antisense reagents are shown in the top panels and RNAi reagents in the bottom panels. Knockdown sites are linearly ordered along the transcript from the 5′- to the 3′-end with different classes of compounds color coded, making it easier to distinguish effects based on reagent type versus location within the target gene. With all sites present on a single plot it becomes even more evident that the ASO reagents performed better for nuclear lncRNAs, RNAi reagents performed better for the cytoplasmic lncRNAs and results were mixed for the dual-localized lncRNAs, with the ASO reagents performing slightly better.

### Combinatorial studies

**Figure 5.**
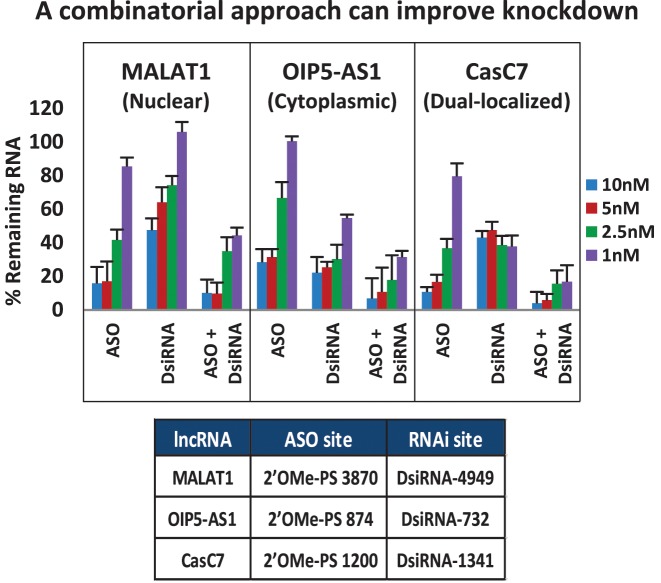
ASO and RNAi combinatorial studies. DsiRNAs and ASOs targeting MALAT1, OIP5-AS1 or CasC7 were transfected in triplicate either independently or together into HeLa cells with Lipofectamine^®^ 2000, and RNA levels were measured by RT-qPCR 24 h later. Data show an average of three experiments. LncRNA levels were calculated using the internal reference genes HPRT and SFRS9, and compared to HeLa cells transfected with 2–3 negative control sequences at the same total dose. Target sites are identified in the table beneath the plots.

Many lncRNAs are not restricted to either the nuclear or cytoplasmic compartment but exist in variable concentrations between the different subcellular locations (16). Simultaneously using more than one knockdown strategy may be more effective than using either method alone for this class of targets. To test whether combinatorial use of antisense and RNAi reagents could improve suppression of lncRNA targets, 2′ OMe-PS ASOs and RNAi reagents were compared for target knockdown efficacy either singly or combined (Figure 5 and Supplemental Figure S10). For MALAT1 (nuclear), OIP5-AS1 (cytoplasmic) and CasC7 (mixed), we observed that combining two RNAi-class reagents had minimal benefit in improving knockdown with the exception of the cytoplasmic lncRNA OIP5-AS1 which had enhanced knockdown efficiency at the lowest dose (Supplemental Figure S10). This was not surprising, as co-transfection of several siRNAs results in competition for RISC entry and usually the most potent siRNA in a group shows a dominant effect (99,100). ASOs do not compete for RNase H1 binding in the same way that siRNAs compete for RISC; they can hybridize to different sites on a target and can show additive knockdown effects. We observed that combining two ASOs slightly increased knockdown at the 1 nM dose for MALAT1 and CasC7 (Supplemental Figure S10). Since antisense and RNAi reagents appear to show maximal activity in different compartments, combining knockdown reagents with different modes of action may have the greatest benefit. It was previously demonstrated that a combination of RNAi and ASO reagents can improve knockdown of an mRNA when targeting different regions of the transcript (64). For the lncRNAs tested in this study, the combination approach improved target knockdown, especially for the lncRNA target CasC7, which is present in both nuclear and cytoplasmic compartments. Similar effects were seen with nuclear NEAT1, cytoplasmic DANCR and the dual-localized TUG1 (data not shown).

### Relative potency of different knockdown methods

**Figure 6.**
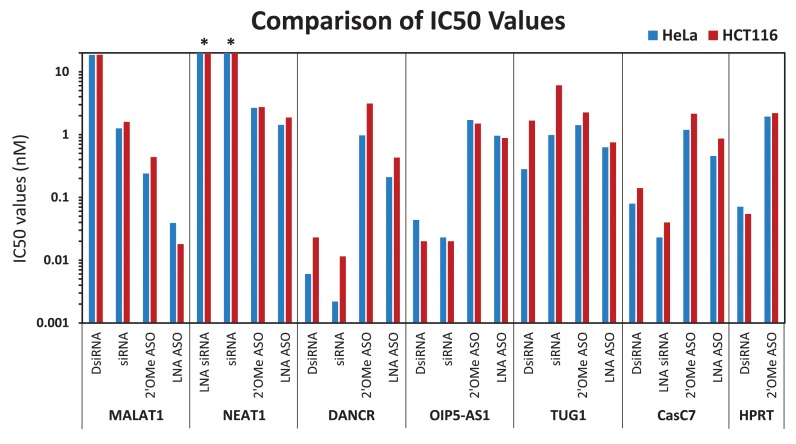
IC50 values of representative knockdown reagents. Two of the most potent ASOs and RNAi reagents were selected for each RNA target and IC50 values (nM) were generated using a 9-point dose response curve in HeLa (blue bars) and HCT116 (red bars) cells. Transfections were performed in triplicate with Lipofectamine^®^ 2000 and RNA levels were measured by RT-qPCR 24 h later. LncRNA levels were calculated using the internal reference genes HPRT and SFRS9, and compared with HeLa cells transfected with 2–3 negative control sequences containing the same chemical modifications at the same doses. IC50 values (nM) were determined using GraphPad PRISM^®^ software. An ‘*’ denotes indeterminate IC50 values (nM) due to ineffective knockdown.

It is desirable to employ knockdown reagents which are potent and have minimal toxicity. SiRNAs have been reported to be ≥100-fold more potent than ‘first generation’ DNA-PS ASOs for RNA knockdown, as evidenced by IC50 values in comparative studies at the same target sites (101–103). ‘Second generation’ modified ASOs can have very high potency, similar to siRNAs (22,103). To allow fair comparisons between reagents, the same dose range was employed for all compounds studied (1–10 nM). In some cases, these doses were well above the IC50 points so the actual potency of those compounds was undefined, requiring additional study at lower doses. The top two performing ASOs and RNAi reagents for six lncRNAs were selected for further study and the actual potency of these 24 compounds was determined in two human cell lines, HeLa and HCT116. Reagents were transfected to establish a 9-point dose response curve, adjusting the dose range employed as needed for each reagent and IC50 values were calculated (Figure 6 and Supplemental Table S2). IC50 values were grossly concordant between the two cells lines, indicating that results are not cell-line specific. For the two nuclear lncRNAs, MALAT1 and NEAT1, IC50 values were lower for the ASOs compared to the RNAi reagents. Alternatively, for the cytoplasmic lncRNAs, DANCR and OIP5-AS1, the RNAi reagents had lower IC50 values than the ASOs. In fact, the lowest IC50 value calculated in this study (2 pM) was for a siRNA targeting DANCR at position 679. TUG1, which has mixed localization, had similar IC50 values for all knockdown reagents. The other dual-localized lncRNA, CasC7, showed lower IC50 values for the two most potent RNAi reagents, even though overall the ASOs were more likely to be able to knockdown CasC7 (Figure 3). We also measured the IC50 values for knockdown of HPRT mRNA using a DsiRNA and 2′ OMe-PS ASO (these reagents were employed throughout the study as the ‘positive control’ sequences used as quality control for transfection efficiency of individual experiments). For these anti-HPRT mRNA reagents, the DsiRNA had a 30–40-fold lower IC50 value than the 2′ OMe-PS ASO.

The RNAi reagent comparison was designed to include sequences optimal for each class of RNAi reagents (DsiRNAs, siRNAs and Silencer^®^ Select siRNAs). Oftentimes, potent siRNA sites do not correlate with potent DsiRNA sites and *vice* *versa* (104–106). To avoid bias by using one selection algorithm over another for all RNAi reagents, the preferred algorithm was used for each class of RNAi reagents. Using these optimally designed RNAi reagents, there was no obvious benefit observed between classes for targeting lncRNAs.

Use of different site selection methods led to different sites being tested for the 2′ OMe-PS ASOs, LNA-PS ASOs, DsiRNAs and siRNAs. To investigate the possibility that structural differences in the lncRNA or protein binding sites consistently impeded the knockdown efficiency of one class of reagent over another, these four classes of reagents were compared at the same six sites in MALAT1. The six sites (LNA-ASO sites from in Figure 1A), were selected by a proprietary Exiqon algorithm that identified locations 16 nt in length for ASO targeting; 2′ OMe-PS 20mers were placed at these sites by extending 2 nt in each direction and the RNAi reagents were extended an extra base for the 21mer siRNAs. The active, ‘diced’ 21mer component of the DsiRNAs were positioned identically to the siRNAs. Compounds were studied for efficacy in MALAT1 knockdown using doses of 10, 1 and 0.1 nM (Supplementary Figure S11). The LNA-PS and 2′ OMe-PS gapmer ASOs showed similar potency at four of the six sites, and the LNA-PS ASOs were more potent at two of the six sites. Therefore the LNA-PS ASOs did show some advantages in potency; however, care must be taken in their use as they also have the highest toxicity (28,107,108). The RNAi reagents were inactive at four of the six sites. For sites 5174 and 7505, the DsiRNA had moderate potency at the 10 nM dose, but never reached the potency level of the ASOs.

Potency of the DNA-PS ASOs and 2′ OMe-PS ASOs was directly compared at the same 12 sites in all seven lncRNAs. A marked advantage for the 2′ OMe-modified compounds was observed (Supplementary Figures S1–S7). However, general knockdown trends were the same between the two classes of ASOs, with good DNA-PS ASO sites also serving as good 2′ OMe-PS ASO sites, which may allow for the less expensive DNA-PS ASOs to be employed for screening ‘walks’ to identify good sites in high value targets with later conversion to a more active 2′ OMe-PS gapmer design.

## DISCUSSION

Antisense and RNAi are both widely used techniques for sequence-specific reduction of cellular RNA expression. They employ distinct mechanisms of action with different protein co-factors and/or effector molecules that have different intracellular distribution patterns. While neither method is restricted to a single subcellular compartment, relative activity can vary between compartments and can affect the ease with which that method can be used to knockdown an RNA target. RNase H-mediated ‘degradative’ antisense relies upon hybridization of an ASO to the RNA target to form an RNA:DNA heteroduplex substrate for RNase H1, which is largely (but not exclusively) found in the nucleus (20–24). The ‘degradative’ arm of RNAi relies upon hybridization of the guide strand of a siRNA to form an RNA:RNA substrate for AGO2 within RISC. Multiple forms of RNAi exist in the nucleus and perform a variety of tasks ranging from heterochromatin formation to transposon regulation; these different functions depend on distinct nuclear RNA Induced Silencing Complexes (RISCs) having different protein components (50). RNAi-mediated degradation of mRNA occurs largely in the cytoplasm, is often associated with the rough endoplasmic reticulum and employs an AGO2-containing RISC (22,45,51–54). The present study examines if relative partitioning of RNase H-active antisense to the nucleus and degradative RNAi to the cytoplasm (or sub-compartments thereof) affects the utility of either method to suppress lncRNA levels.

Targeting mRNAs is somewhat simplified by the single purpose of protein coding transcripts, which are made in the nucleus and translated to protein in the cytoplasm. Hence, RNase H active-ASOs can be used to trigger degradation of nascent pre-mRNA transcripts in the nucleus and siRNAs can be used to trigger degradation of mature mRNAs in the cytoplasm; use of either method serves to prevent translation of the targeted mRNA. Although antisense methods were the first gene knockdown technology developed, RNAi has become the dominant technology employed in research applications, largely due to its ease of use. Efforts to develop algorithms to predict effective ASO sites have not had the same success as for RNAi, probably due to their different mechanisms of action. ASO activity relies upon hybridization of the single-stranded oligonucleotide to the target RNA, which is highly dependent upon both the folded structure of the RNA and the presence of RNA-binding proteins that may limit access. Tertiary structure and protein binding are difficult to predict, particularly for longer RNAs of the type studied herein. ASO site selection was done by combining RNA folding prediction, analysis of oligonucleotide sequence properties and a cross-reactivity search to help ensure specificity (for the DNA-PS and 2′ OMe-PS sites) or by Exiqon using proprietary methods (for the LNA-PS sites). RNAi, on the other hand, relies upon a protein effector complex (RISC) that evolved to mediate gene regulation by endogenous dsRNAs and seems to facilitate interaction between the siRNA and the RNA target. Algorithm development for siRNA site selection has been very successful (109), reducing (but not eliminating) the need for empiric testing of reagents. Site selection for the RNAi reagents used in the present study employed a DsiRNA-specific support vector machine (SVM) algorithm (Integrated DNA Technologies), an siRNA algorithm (Dharmacon, GE Healthcare) (110,111) and an siRNA SVM algorithm (Ambion, Thermo Fisher Scientific) (112). The best target sites within the same gene vary between ASOs and siRNAs (113), and, not surprisingly, different sites were selected by the different algorithms employed.

Targeting lncRNAs is complicated by their wide range of functions and subcellular distribution patterns. LncRNAs play many different roles in the cell, including, for example, association with chromatin or with mRNA, or binding miRNAs or proteins (‘sponge’ effects). Some lncRNAs reside mainly in the nucleus, some reside primarily in the cytoplasm, others are present in both compartments, while yet others concentrate in specific subcellular organelles. It is, therefore, not surprising that lncRNAs might be more difficult to suppress than mRNAs or that greater challenges would be encountered when trying to use the different gene knockdown techniques available.

The present study observed that achieving high levels of knockdown of nuclear lncRNAs was easier using antisense methods while knockdown of cytoplasmic lncRNAs was easier using RNAi methods. Consistent with this observation, antisense methods are thought to be particularly effective in suppressing nuclear-retained RNAs, including lncRNAs such as MALAT1 (24,114). Interestingly, the results presented here suggest that antisense knockdown of cytoplasmic lncRNAs may be more effective than RNAi knockdown of nuclear lncRNAs (Figures 4 and 6). This implies that the level of RNase H1 activity may be higher in the cytoplasm than was previously appreciated. A recent study demonstrated that the rate-limiting step of mRNA degradation following transfection of ASOs directly related to the amount of RNase H1 present and further showed that RNase H1 activity was present in both the cytoplasm and the nucleus; although the relative rate of target RNA cleavage was faster in the nucleus, ASO-directed RNA degradation was effective in both compartments (115).

It is interesting that the cytoplasmic targets DANCR and OIP5-AS1 both associate with 40S/60S ribosomal subunits, placing them in proximity to known sites for high RNAi activity (15,51). This may account for the very high potency observed for the RNAi reagents for these targets (Figure 2A and B). The high success rate of the RNAi reagents designed to target cytoplasmic lncRNAs also indicates that the existing siRNA and DsiRNA design algorithms (which were trained using mRNA targeting data) are also effective at finding active sites to target lncRNAs.

The results reported and discussed herein employed measurements of RNA levels taken 24 h post-transfection and, therefore, assess the immediate effects seen on degradation of target molecules present in accessible compartments. Similar results were observed if measurements were taken 48 h post-transfection (data not shown); however, longer incubation periods were not studied. Since transcription occurs in the nucleus, antisense methods, which utilize nuclear RNase H1, should eventually be effective at suppressing all lncRNAs, regardless of where the mature lncRNA product later accumulates in the cell. The time course of this knockdown will vary with the turnover rates for each lncRNA species.

The present study employed two RT-qPCR assays to measure levels of each lncRNA. One assay was located toward the 5′-end and the other toward the 3′-end of the target. Using two (or more) assays is needed to help rule out assay-related artifacts, such as false positive results due to interference of the oligonucleotide-based knockdown reagents with reverse transcription or qPCR (116). Also, target cleavage is no guarantee of subsequent rapid RNA degradation. In many cases, rapid degradation occurs; however, occasionally, ‘retained fragments’ persist that can be detected as a false negative result by a RT-qPCR assay that lies within the retained fragment (our unpublished observation). In fact, such fragments have been shown to retain function (117). Both RT-qPCR assays for each of the lncRNA targets studied showed grossly concordant results (Supplementary Figure S8 and data not shown), suggesting that even for the longer lncRNAs studied (MALAT1, 8.7 kb; and CasC7, 9.3 kb), the transcripts were degraded after cleavage. However, for lncRNAs that are much longer (e.g. Airn is ∼118 kb), nuclease degradation after cleavage via ASOs or RNAi might not go to completion, especially if the lncRNA is complexed with proteins or includes highly structured domains. For mRNAs, even if complete degradation does not immediately follow cleavage at an ASO or siRNA site, translation of the surviving RNA species would produce a truncated protein (if the target site was positioned within the coding region), which may result in a non-functional product. The varied spectrum of lncRNA function increases the likelihood of residual activity remaining in even a small retained fragment if an important miRNA-binding site, protein binding site or other important structural feature of that RNA survived intact. Simultaneous targeting of multiple sites could reduce the risk of retaining functional fragments. It may also be possible to block lncRNA function without degradation. For example, steric-blocking ASOs made using 2′ OMe RNA and LNA residues (containing no DNA, eliminating RNase H-active degradative pathways) targeting natural antisense transcripts (NATs, a form of lncRNAs) have been shown to increase levels of brain-derived neurotrophic factor (BDNF) by blocking suppression mediated by the NATs (41). A steric-blocking ASO approach will, however, require more extensive empirical testing (perhaps even ‘gene walks’) to identify the precise sites at which ASO binding can block lncRNA function. This approach also offers the possibility to interfere with one function of a lncRNA without altering other functions (e.g. block a binding site for one miRNA or one protein, but not neighboring binding sites for different molecules which may co-exist on a single lncRNA species).

Different methods can be used to suppress expression of lncRNAs. Due to their varied function and subcellular distribution, antisense methods (for nuclear species) or RNAi methods (for cytoplasmic species) may show better performance. When the distribution of a lncRNA is unknown, antisense methods may be more reliable for achieving knockdown if only one approach is employed. Combined use of antisense with RNAi methods can lead to better knockdown than use of either method alone, especially for lncRNA targets that are distributed throughout the cell.

## Supplementary Material

SUPPLEMENTARY DATA
