# Supplementary material for: Cellular localization of long non-coding RNAs affects silencing by RNAi more than by antisense oligonucleotides
Source: Nucleic Acids Res. 2015 Nov 17;44(2):863–77. doi: 10.1093/nar/gkv1206 (PMC4737147; doi:10.1093/nar/gkv1206)
Supplement: SUPPLEMENTARY DATA [file supp_44_2_863__index.html]

Cellular localization of long non-coding RNAs affects silencing by RNAi more than by antisense oligonucleotides — SUPPLEMENTARY DATA 

# Cellular localization of long non-coding RNAs affects silencing by RNAi more than by antisense oligonucleotides

## SUPPLEMENTARY DATA

- SUPPLEMENTARY DATA
- SUPPLEMENTARY DATA
