# Supplementary material for: Cellular localization of long non-coding RNAs affects silencing by RNAi more than by antisense oligonucleotides
Source: Nucleic Acids Res. 2015 Nov 17;44(2):863–77. doi: 10.1093/nar/gkv1206 (PMC4737147; doi:10.1093/nar/gkv1206)
Supplement: SUPPLEMENTARY DATA [file supp_gkv1206_nar-01025-y-2015-File009.pdf]

Supplemental Figures:

Figure S1

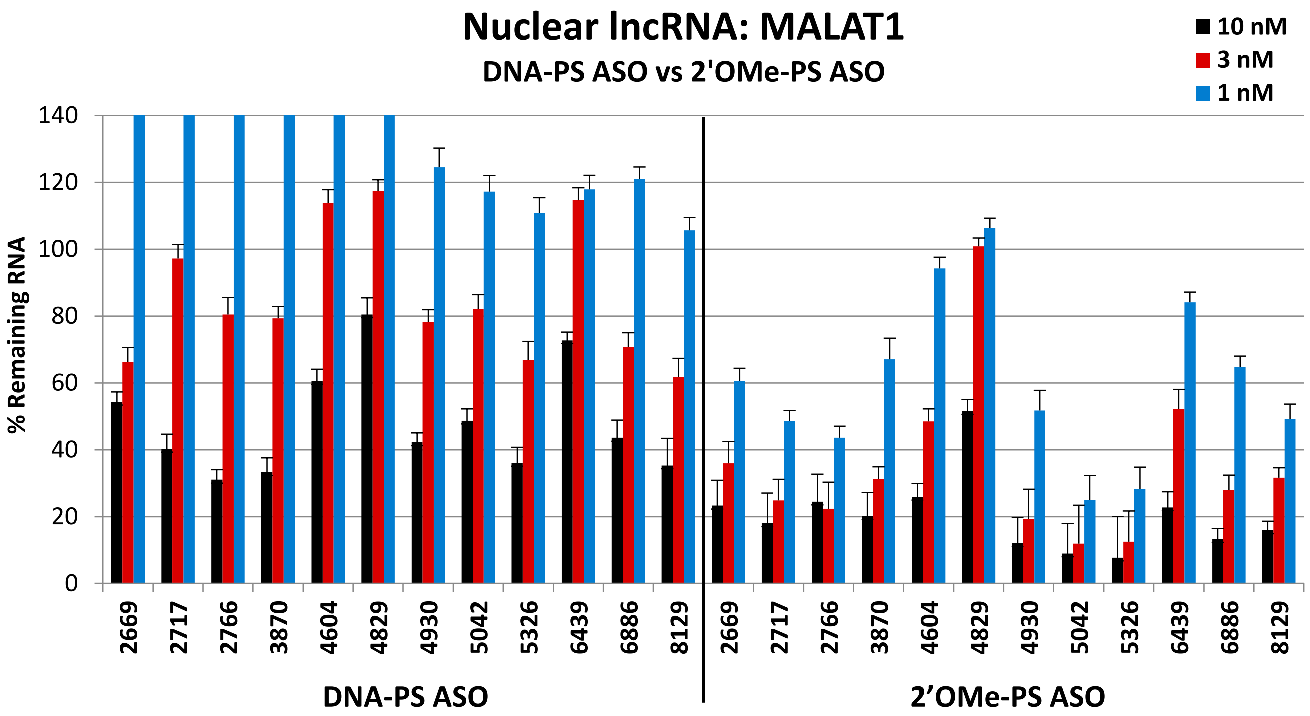

Figure S1: Potency comparison between DNA-PS and 2'OMe-PS ASOs for MALAT1. DNA-PS or 2'OMe-PS ASOs at the same sites targeting MALAT1 were transfected in triplicate into HeLa cells with Lipofectamine® 2000, and RNA levels were measured by RT-qPCR 24 hrs later. Data shows an average of two or more experiments (e.g., at least 6 independent transfections). LncRNA levels were calculated using the internal reference genes HPRT and SFRS9, and compared against HeLa cells transfected with 2-3 negative control sequences containing the same chemical modifications at the same dose. Site locations in the target are indicated on the x-axis, and organized 5' to 3' along each target for each class of knockdown reagents.

Figure S2

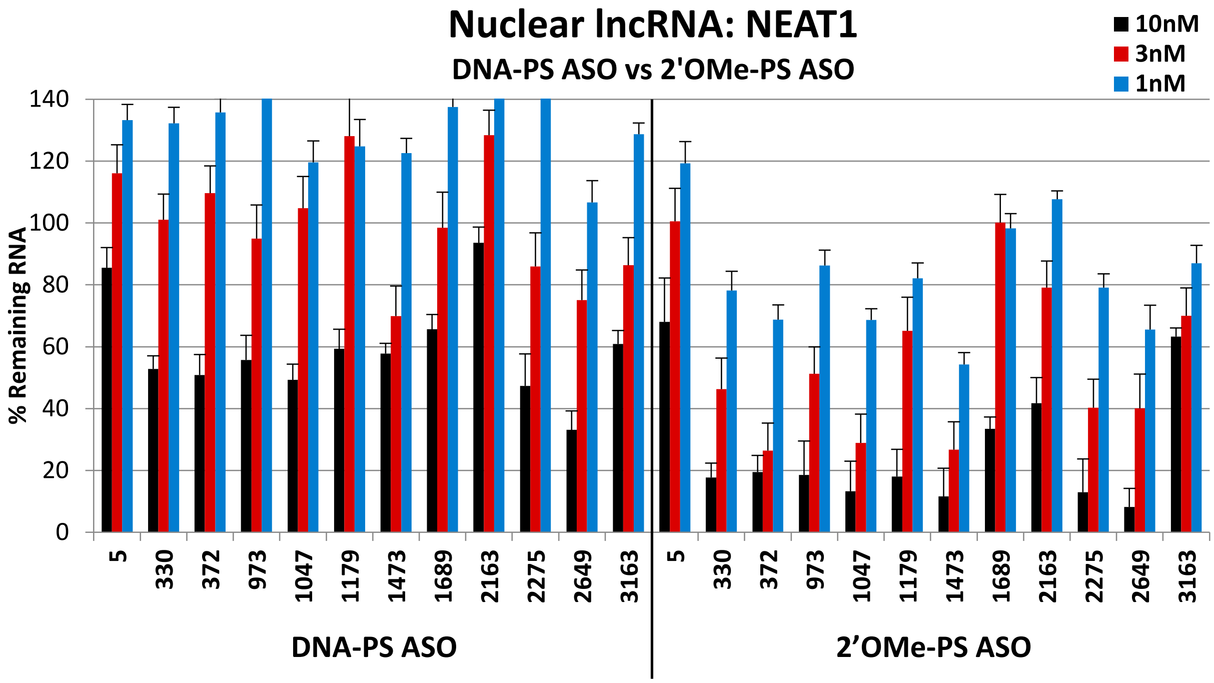

Figure S2: Potency comparison between DNA-PS and 2'OMe-PS ASOs for NEAT1.
DNA-PS or 2'OMe-PS ASOs at the same sites targeting NEAT1 were transfected in triplicate into HeLa cells with Lipofectamine® 2000, and RNA levels were measured by RT-qPCR 24 hrs later. Data shows an average of two or more experiments (e.g., at least 6 independent transfections). LncRNA levels were calculated using the internal reference genes HPRT and SFRS9, and compared against HeLa cells transfected with 2-3 negative control sequences containing the same chemical modifications at the same dose. Site locations in the target are indicated on the x-axis, and organized 5' to 3' along each target for each class of knockdown reagents.

Figure S3

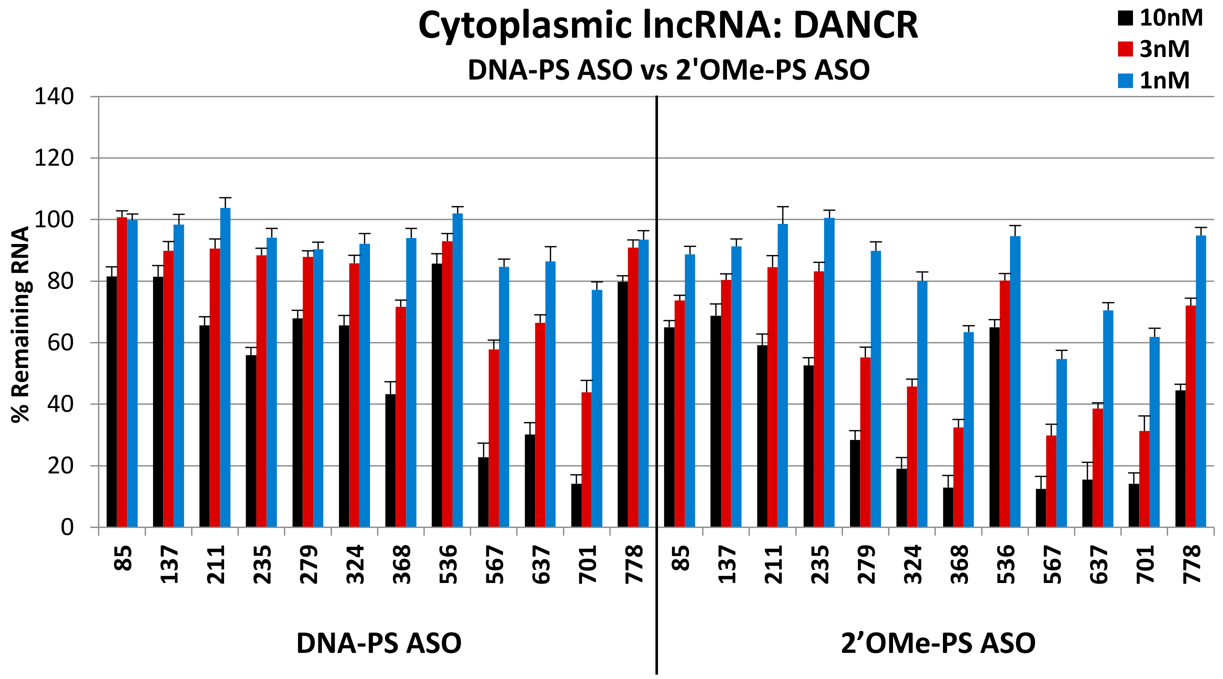

Figure S3: Potency comparison between DNA-PS and 2'OMe-PS ASOs for DANCR. DNA-PS or 2'OMe-PS ASOs at the same sites targeting DANCR were transfected in triplicate into HeLa cells with Lipofectamine® 2000, and RNA levels were measured by RT-qPCR 24 hrs later. Data shows an average of two or more experiments (e.g., at least 6 independent transfections). LncRNA levels were calculated using the internal reference genes HPRT and SFRS9, and compared against HeLa cells transfected with 2-3 negative control sequences containing the same chemical modifications at the same dose. Site locations in the target are indicated on the x-axis, and organized 5' to 3' along each target for each class of knockdown reagents.

Figure S4

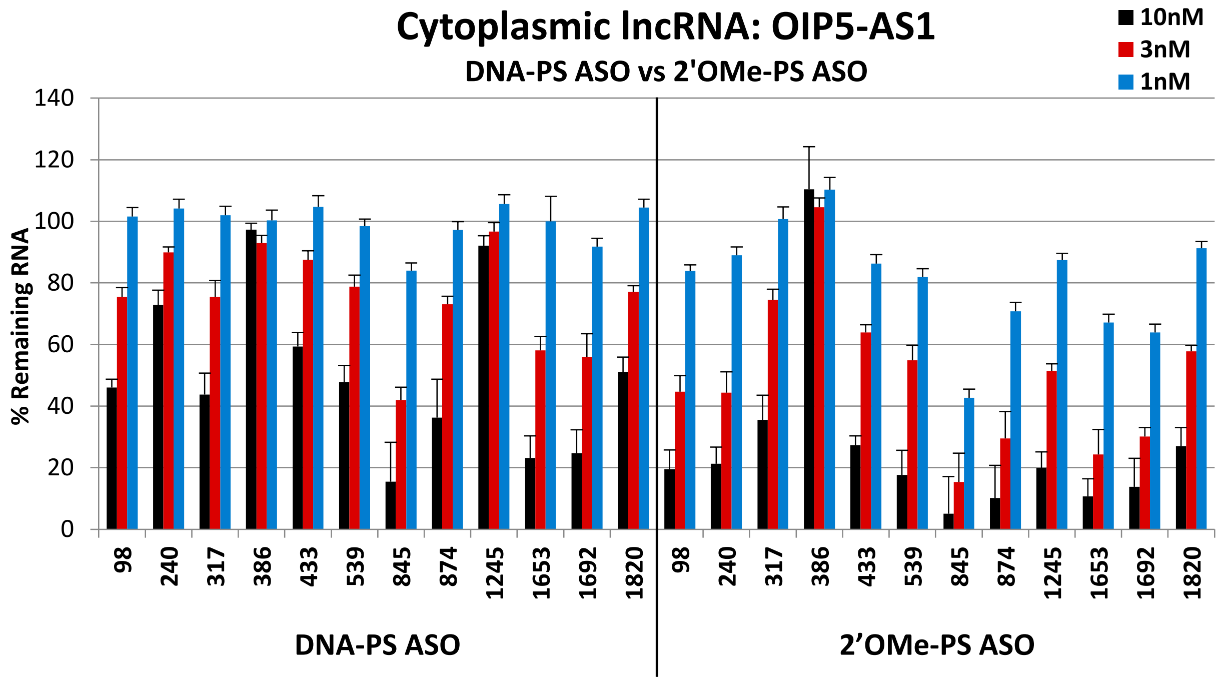

Figure S4: Potency comparison between DNA-PS and 2'OMe-PS ASOs for OIP5-AS1. DNA-PS or 2'OMe-PS ASOs at the same sites targeting OIP5-AS1 were transfected in triplicate into HeLa cells with Lipofectamine® 2000, and RNA levels were measured by RT-qPCR 24 hrs later. Data shows an average of two or more experiments (e.g., at least 6 independent transfections). LncRNA levels were calculated using the internal reference genes HPRT and SFRS9, and compared against HeLa cells transfected with 2-3 negative control sequences containing the same chemical modifications at the same dose. Site locations in the target are indicated on the x-axis, and organized 5' to 3' along each target for each class of knockdown reagents.

Figure S5

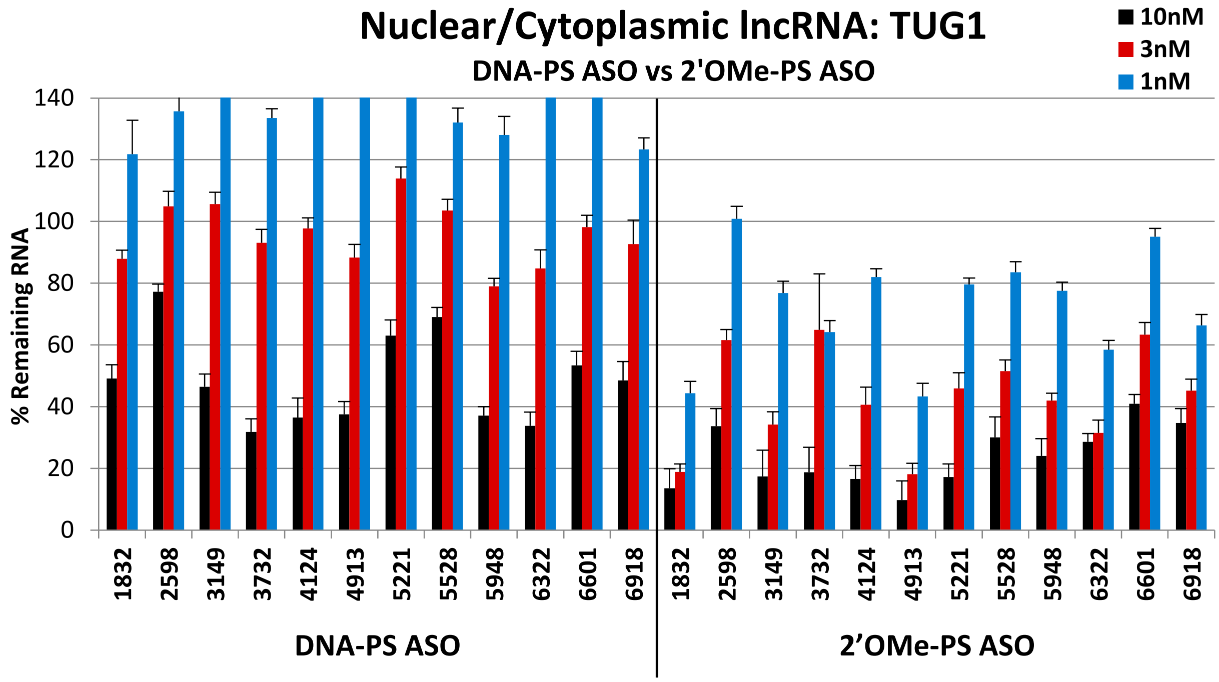

Figure S5: Potency comparison between DNA-PS and 2'OMe-PS ASOs for TUG1.
DNA-PS or 2'OMe-PS ASOs at the same sites targeting TUG1 were transfected in triplicate into HeLa cells with Lipofectamine® 2000, and RNA levels were measured by RT-qPCR 24 hrs later. Data shows an average of two or more experiments (e.g., at least 6 independent transfections). LncRNA levels were calculated using the internal reference genes HPRT and SFRS9, and compared against HeLa cells transfected with 2-3 negative control sequences containing the same chemical modifications at the same dose. Site locations in the target are indicated on the x-axis, and organized 5' to 3' along each target for each class of knockdown reagents.

Figure S6

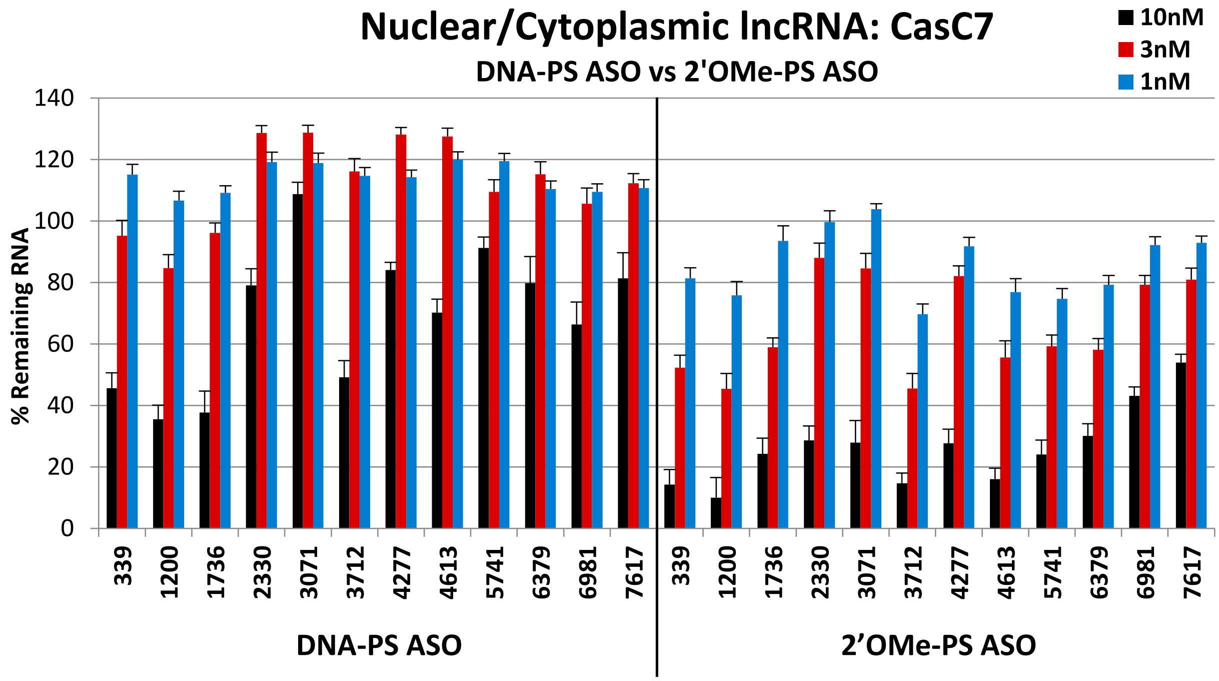

Figure S6: Potency comparison between DNA-PS and 2’OMe-PS ASOs for CasC7.
DNA-PS or 2’OMe-PS ASOs at the same sites targeting CasC7 were transfected in triplicate into HeLa cells with Lipofectamine® 2000, and RNA levels were measured by RT-qPCR 24 hrs later. Data shows an average of two or more experiments (e.g., at least 6 independent transfections). LncRNA levels were calculated using the internal reference genes HPRT and SFRS9, and compared against HeLa cells transfected with 2-3 negative control sequences containing the same chemical modifications at the same dose. Site locations in the target are indicated on the x-axis, and organized 5’ to 3’ along each target for each class of knockdown reagents.

Figure S7

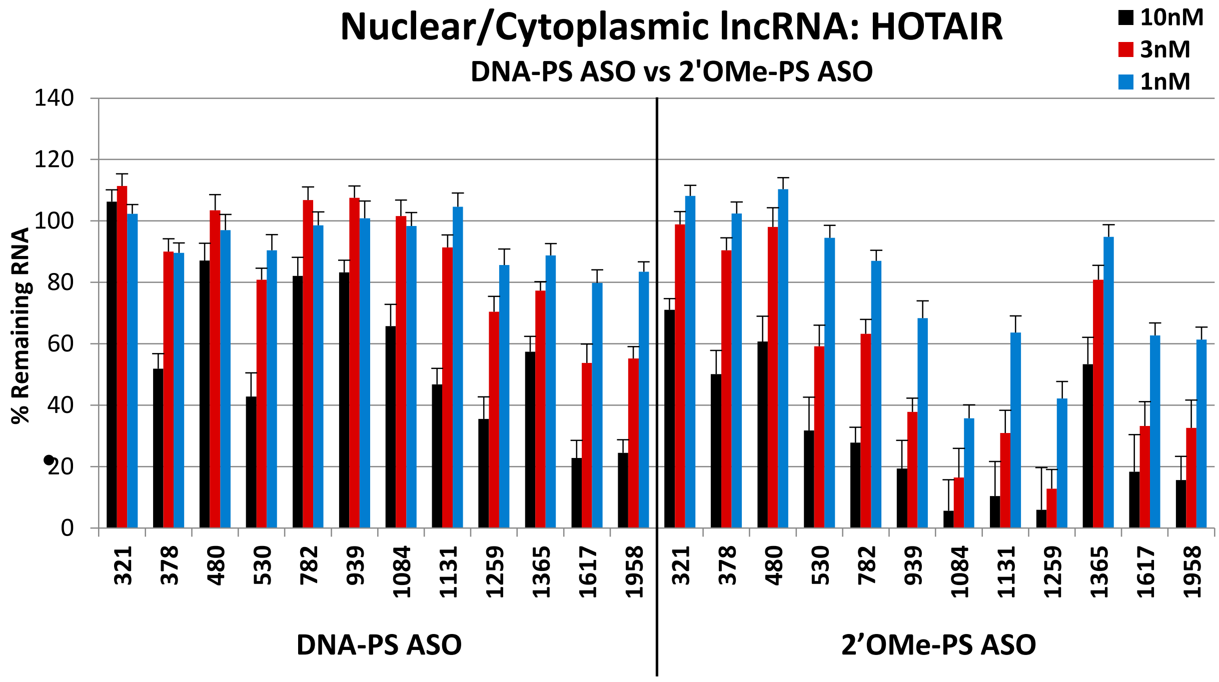

Figure S7: Potency comparison between DNA-PS and 2'OMe-PS ASOs for HOTAIR.
DNA-PS or 2'OMe-PS ASOs at the same sites targeting HOTAIR were transfected in triplicate into HeLa cells with Lipofectamine® 2000, and RNA levels were measured by RT-qPCR 24 hrs later. Data shows an average of two or more experiments (e.g., at least 6 independent transfections). LncRNA levels were calculated using the internal reference genes HPRT and SFRS9, and compared against HeLa cells transfected with 2-3 negative control sequences containing the same chemical modifications at the same dose. Site locations in the target are indicated on the x-axis, and organized 5' to 3' along each target for each class of knockdown reagents.

Figure S8

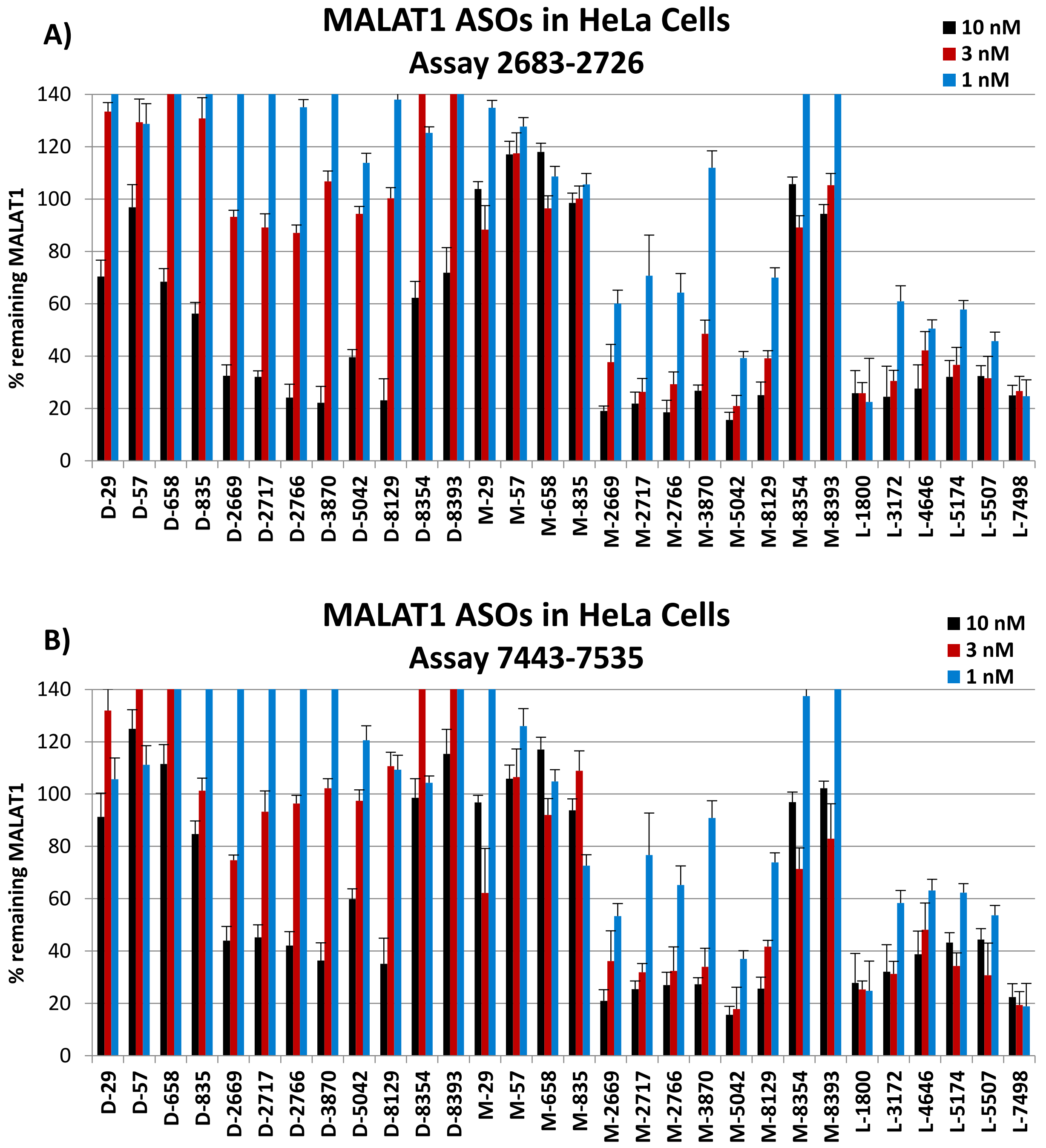

Figure S8: An example of qPCR 5' and 3' assay concordance.
ASOs targeting MALAT1 were transfected in triplicate into HeLa cells with Lipofectamine® 2000, and RNA levels were measured by RT-qPCR 24 hrs later by multiplexing A) a qPCR assay positioned at 2683-2726 and B) a qPCR assay positioned at 7443-7535. LncRNA levels were calculated using the internal reference genes HPRT and SFRS9, and compared against HeLa cells transfected with 2-3 negative control sequences containing the same chemical modifications at the same dose. Site locations in the target are indicated on the x-axis, and organized 5' to 3' along each target for each class of knockdown reagents. D = DNA-PS, M = 2'OMe-PS gapmer and L = LNA-PS gapmer.

Figure S9

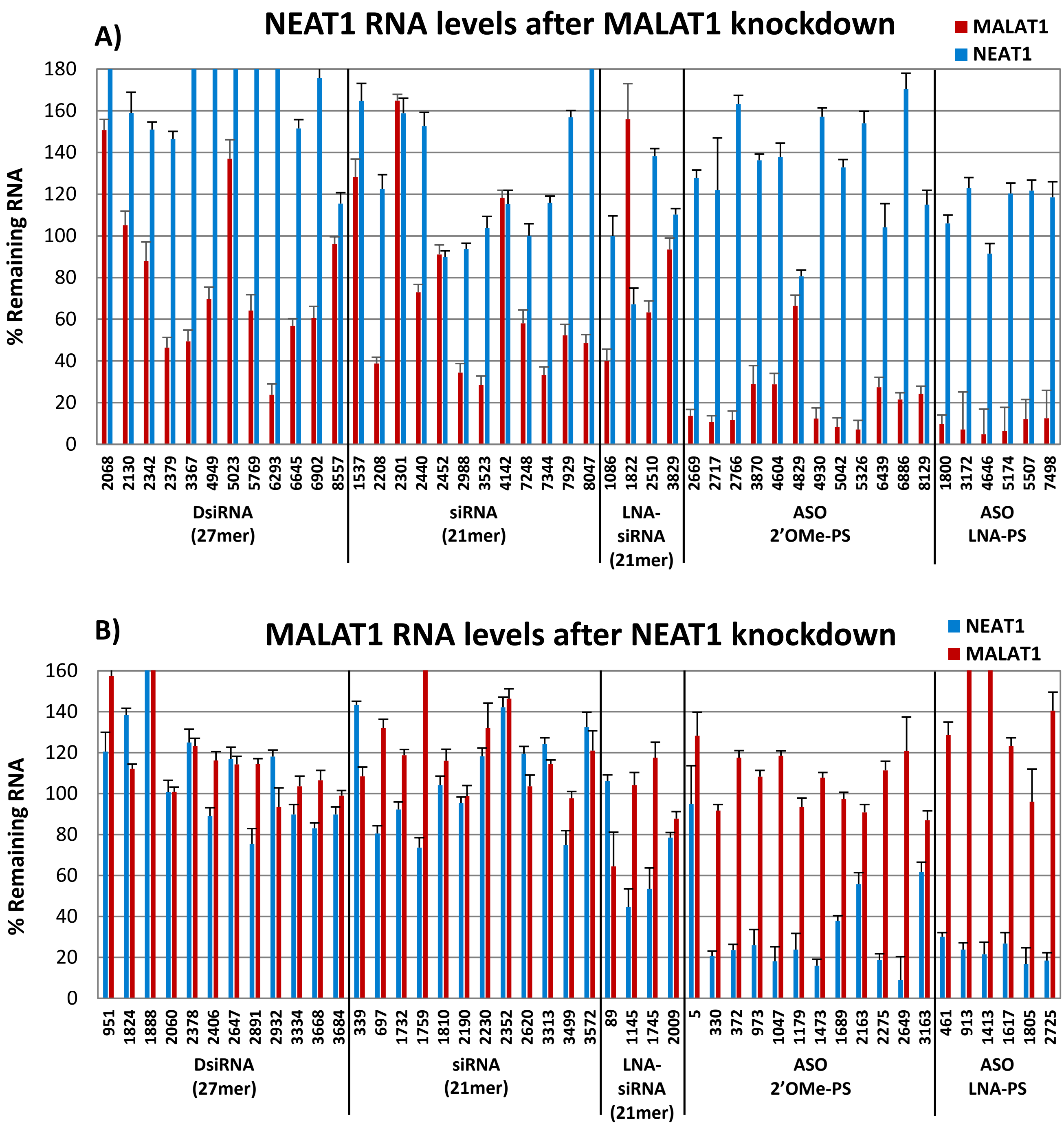

Figure S9: Specificity of MALAT1 and NEAT1 knockdown.
DsiRNAs, siRNAs and ASOs targeting A) MALAT1 or B) NEAT1 were transfected in triplicate into HeLa cells with Lipofectamine® 2000, and RNA levels were measured by RT-qPCR 24 hrs later. Data shown represent an average of triplicate transfections. MALAT1 and NEAT1 levels were calculated using the internal reference genes HPRT and SFRS9, and compared with HeLa cells transfected with 2-3 negative control sequences containing the same chemical modifications at the same doses. Site locations of the knockdown reagents are indicated on the x-axis and are organized 5' to 3' along each target for each class of knockdown reagent.

Figure S10

A)

lncRNA	ASO #1 site	ASO #2 site	DsiRNA site	siRNA site
MALAT1	2'OMe-PS 3870	2'OMe-PS 2766	DsiRNA 4949	siRNA 2988
OIP5-AS1	2'OMe-PS 874	2'OMe-PS 1653	DsiRNA 732	siRNA 665
CasC7	2'OMe-PS 1200	2'OMe-PS 3713	DsiRNA 1341	siRNA 5946

B)

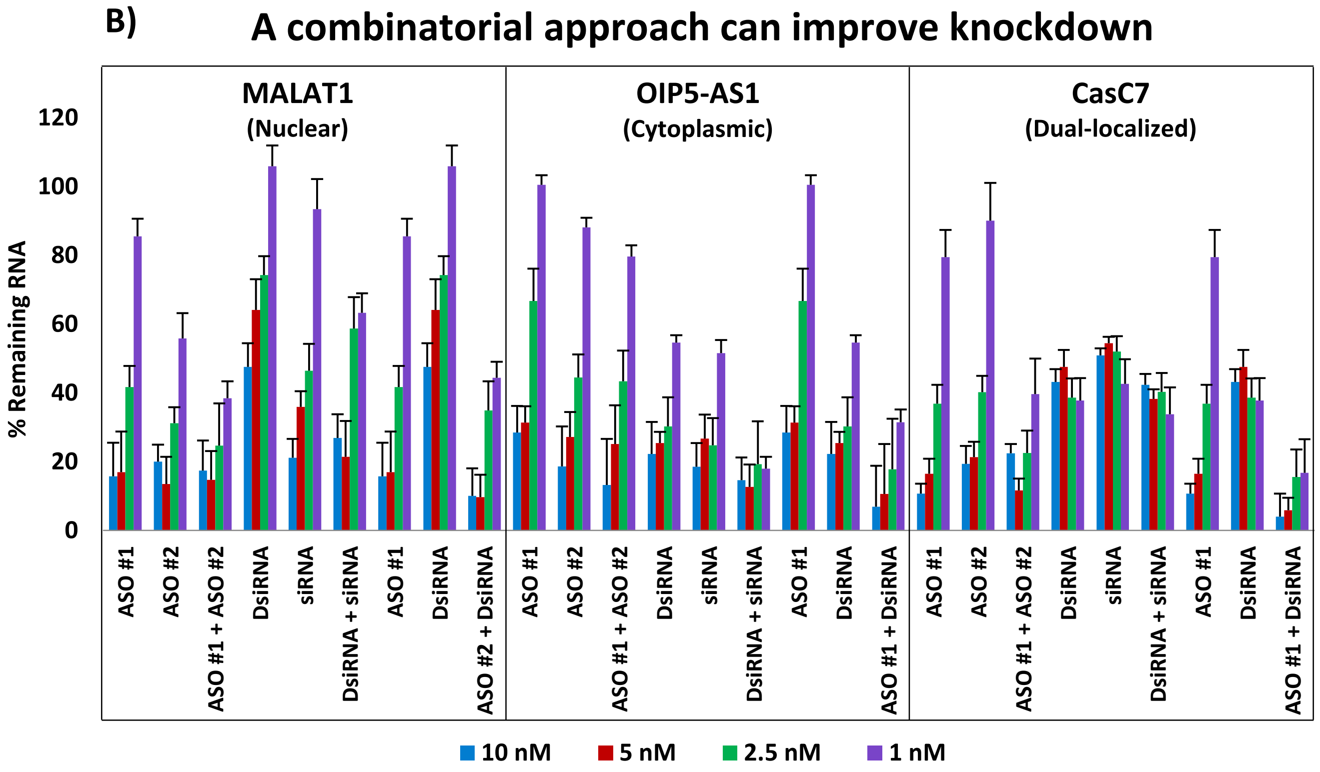

Figure S10: A combinatorial approach can improve knockdown.
2'OMe-PS ASOs, DsiRNAs and siRNAs were transfected alone or cotransfected in combination with another knockdown reagent. A) Target site locations of each knockdown reagent. B) Transfections were performed in triplicate into HeLa cells with Lipofectamine® 2000, and RNA levels were measured by RT-qPCR 24 hrs later. LncRNA levels were calculated using the internal reference genes HPRT and SFRS9, and compared against HeLa cells transfected with 2-3 negative control sequences at the same total dose.

Figure S11

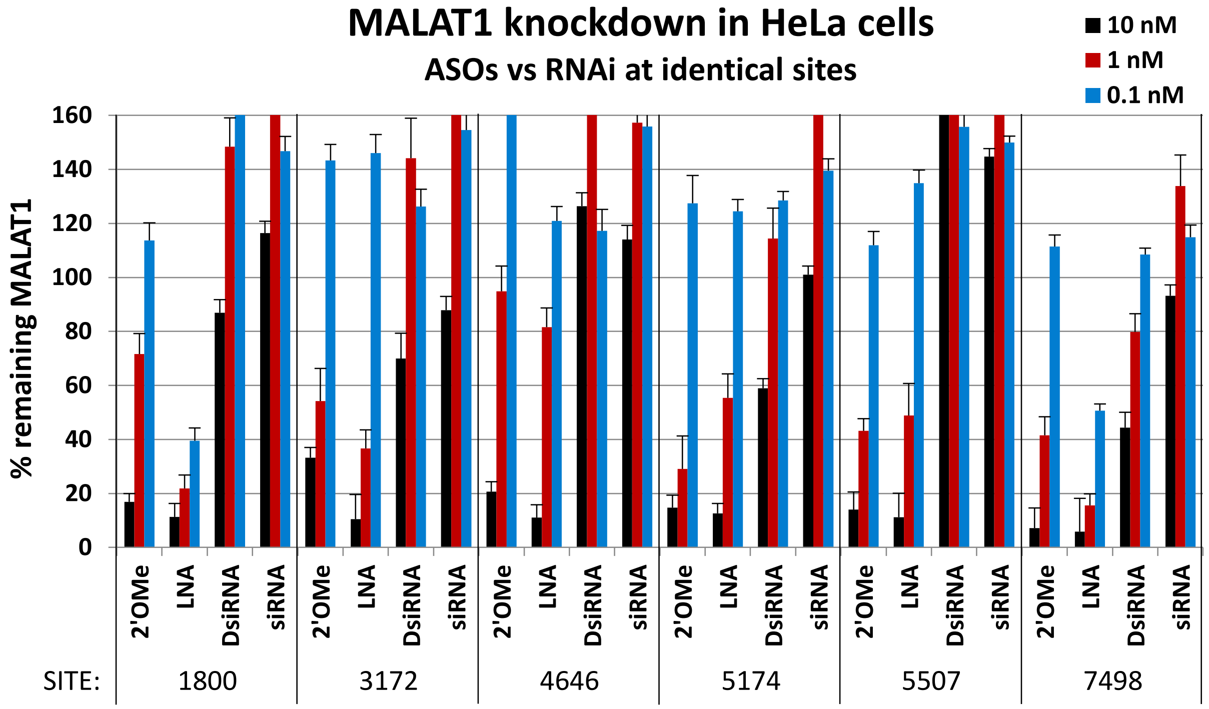

Figure S11: Potency comparison between ASOs and RNAi reagents targeting MALAT1 at identical sites.

2’OMe-PS gapmer and LNA-PS gapmer ASOs were compared with DsiRNAs and siRNAs for potency at the same sites targeting MALAT1. All knockdown reagents were transfected in triplicate into HeLa cells with Lipofectamine® 2000, and RNA levels were measured by RT-qPCR 24 hrs later. LncRNA levels were calculated using the internal reference genes HPRT and SFRS9, and compared against HeLa cells transfected with 2-3 negative control sequences containing the same chemical modifications at the same dose. Site locations in the target are indicated on the x-axis.

Supplemental Table 2

			IC50 (nM)	
**Target**	**Localization**	**Reagent**	**HeLa**	**HCT116**
MALAT1	nuclear	DsiRNA-6928	18.49	18.66
MALAT1	nuclear	siRNA-2988	1.26	1.59
MALAT1	nuclear	2OMe ASO-5042	0.24	0.44
MALAT1	nuclear	LNA ASO-1800	0.04	0.02
NEAT1	nuclear	LNA siRNA-1745	Indeterminate	Indeterminate
NEAT1	nuclear	siRNA-3499	Indeterminate	Indeterminate
NEAT1	nuclear	2OMe ASO-1473	2.66	2.74
NEAT1	nuclear	LNA ASO-1805	1.42	1.87
DANCR	cytoplasmic	DsiRNA-665	0.01	0.02
DANCR	cytoplasmic	siRNA-679	0.002	0.01
DANCR	cytoplasmic	2OMe ASO-567	0.97	3.13
DANCR	cytoplasmic	LNA ASO-703	0.21	0.43
OIP5-AS1	cytoplasmic	DsiRNA-1419	0.04	0.02
OIP5-AS1	cytoplasmic	siRNA-1756	0.02	0.02
OIP5-AS1	cytoplasmic	2OMe ASO-845	1.70	1.49
OIP5-AS1	cytoplasmic	LNA ASO-1367	0.96	0.88
TUG1	mixed	DsiRNA-1724	0.28	1.67
TUG1	mixed	siRNA-1700	0.98	6.10
TUG1	mixed	2OMe ASO-4913	1.41	2.26
TUG1	mixed	LNA ASO-5140	0.63	0.75
CasC7	mixed	DsiRNA 5852	0.08	0.14
CasC7	mixed	LNA siRNA-775	0.02	0.04
CasC7	mixed	2OMe ASO-3713	1.19	2.14
CasC7	mixed	LNA ASO-910	0.45	0.86
HPRT mRNA	mixed	DsiRNA Pos Cont	0.07	0.05
HPRT mRNA	mixed	2OMe ASO Pos Cont	1.93	2.19

IC50 values calculated from 9 doses in HeLa and HCT116 cells with GraphPad Prism® 6.0 software using the non-linear regression formula: log (inhibitor) versus normalized response – variable slope. IC50 values indicated as “indeterminate” indicate reagents in which potency levels were too low for calculations.
